# Numerical comparison of exhaust plume flow behaviors of small monopropellant and bipropellant thrusters

**DOI:** 10.1371/journal.pone.0176423

**Published:** 2017-05-08

**Authors:** Kyun Ho Lee

**Affiliations:** Department of Aerospace Engineering, Sejong University, Seoul, Republic of Korea; North China Electric Power University, CHINA

## Abstract

In general, a space propulsion system has a crucial role in the normal mission operations of a spacecraft. Depending on the types and number of propellants, a monopropellant and a bipropellant thrusters are mostly utilized for low thrust liquid rocket engines. As the plume gas flow exhausted from these small thrusters expands freely in a vacuum space environment along all directions, adverse effects of the plume impingement onto the spacecraft surfaces can dramatically reduce the function and performance of a spacecraft. Thus, the purpose of the present study is to investigate and compare the major differences of the plume gas flow behaviors numerically between the small monopropellant and bipropellant thrusters. To ensure efficient numerical calculations, the whole physical domain was divided into three different subdomains depending on the flow conditions, and then the appropriate numerical methods were combined and applied for each subdomain sequentially. With the present analysis results, the plume gas behaviors including the density, the overall temperature and the separation of the chemical species are compared and discussed between the monopropellant and the bipropellant thrusters. Consequently, the present results are expected to provide useful information on selecting the appropriate propulsion system, which can be very helpful for actual engineers practically during the design process.

## Introduction

**Fig 1:**
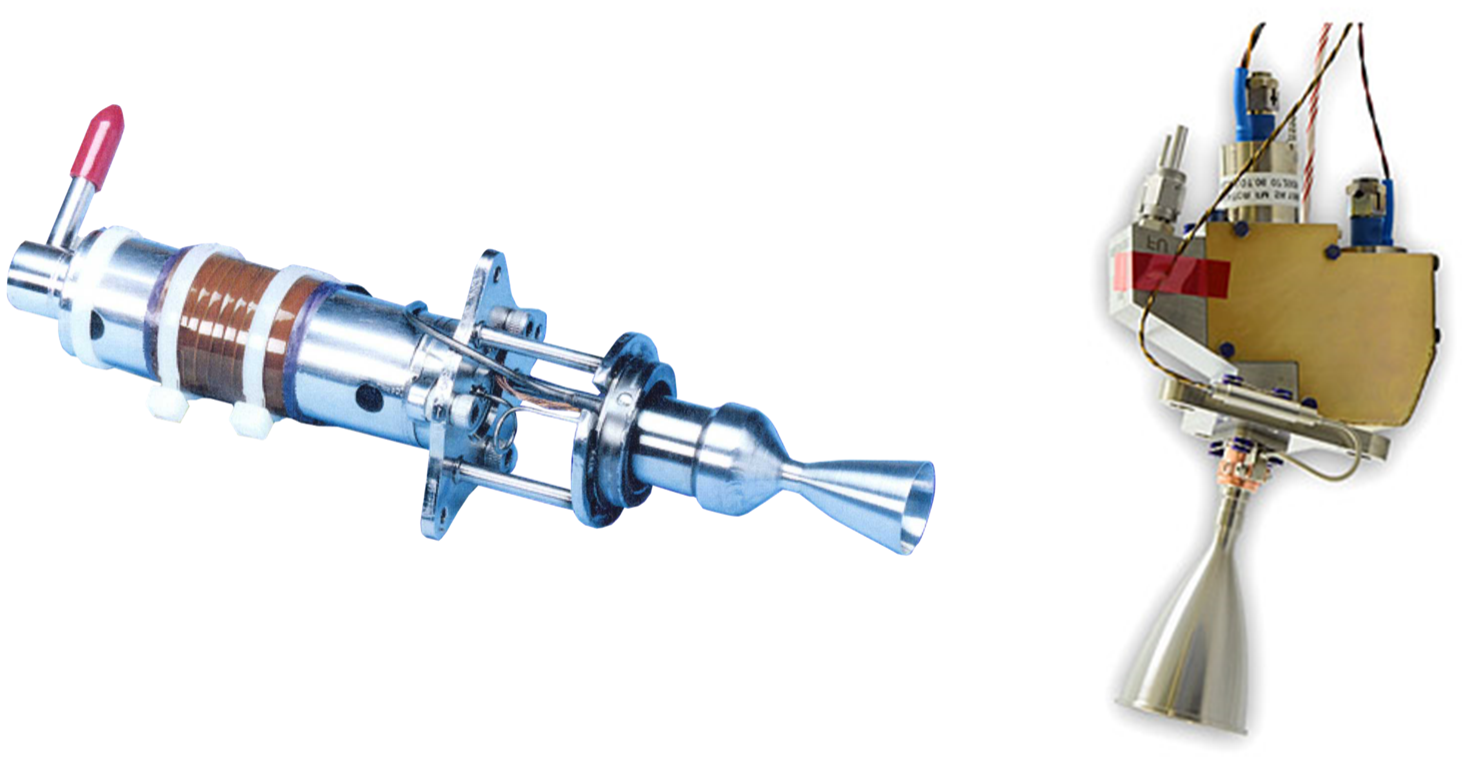
Examples of small monopropellant and bipropellant thrusters. (A) Monopropellant thruster (Hydrazine propellant) [1]. (B) Bipropellant thruster (MMH-NTO propellant) [2].

**Fig 2:**
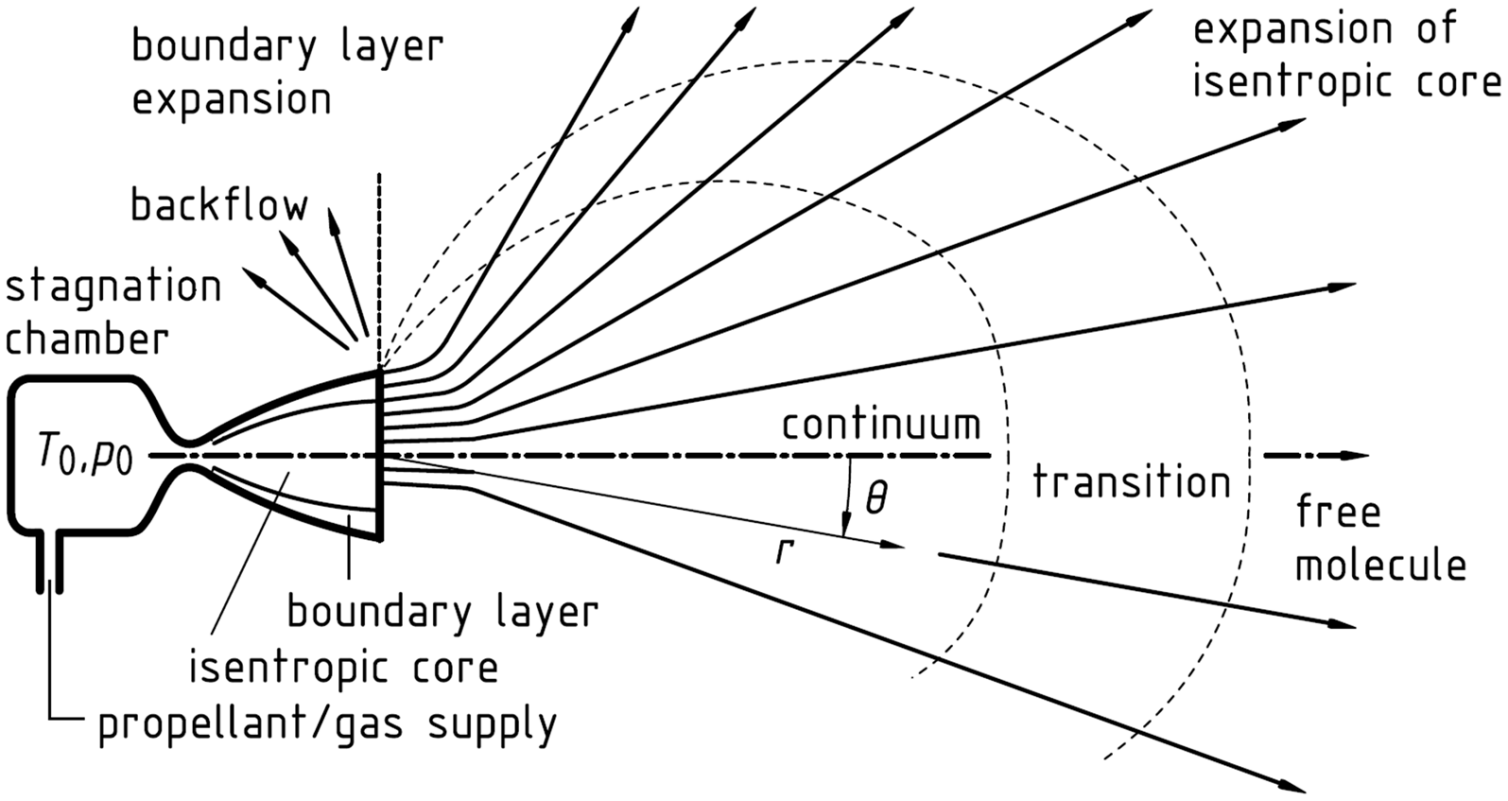
Typical plume flow regimes of thruster in vacuum [3,4].

A space propulsion system has a crucial role in the normal mission operations of a spacecraft by controlling its attitude and maneuver. Depending on the liquid propellant types, a low thrust liquid rocket engine can be classified as two major categories such as monopropellant and bipropellant thrusters in Fig 1 [1,2]. A major difference of these thrusters is that the monopropellant thruster requires only a single fuel which decomposes into hot gas when properly catalyzed while a fuel and oxidizer are split and fed separately into a combustion chamber for a bipropellant thruster. To provide highly precise thrust, this low thrust liquid rocket engine, called a small thruster, converts chemical reaction energy of the liquid propellants into kinetic energy of a high-temperature and high-pressure combustion gas flow (plume gas flow) as the gas is accelerated through a nozzle. But this hot plume gas diffuses in all directions under a vacuum space environment as seen in Fig 2 [3,4], unwanted influences on the spacecraft such as a disturbing force/torque, excessive heat load, and serious contamination of sensitive components can be occurred by the plume gas impingement [3,4]. Thus, one of the major issues when using the thruster involves the accurate assessment and reduction of undesirable effects caused by the plume flow at the design phase of the spacecraft because these effects can dramatically reduce the function and performance of the spacecraft [3].

Therefore, various methods for a numerical simulation have been developed remarkably to predict the physical characteristics of the plume flowfields with the improvement in computation performance rather than using experimental approaches because of several difficulties and complexities to simulate the influences of the thruster firing under high vacuum conditions in a ground facility [3]. Among several methods, the Direct Simulation Monte Carlo (DSMC) method [5,6] has been mostly employed to analyze the plume flowfields in the vacuum because it can accurately simulate a rarefied transition regime and a free molecular flow at the far field from the thruster nozzle exit as shown in Fig 2 [3,4] by solving the Boltzmann equation with statistical techniques. Therefore, numerous studies have been reported recently which investigated the exhaust plume flow phenomena for the small liquid propellant thrusters with the DSMC method [7–15]. In 1980’s, Trinks et al. have compared analytical and experimental results of the small bipropellant thruster [7], and Furlani et al. introduced a parallel algorithm to improve the DSMC method [8]. From 1990’s, the continuum method and the DSMC method have been combined to simulate a continuum-rarefied flow together by several researchers [9–11]. In recent, Tang et al. introduced a possessing adaptive-interface and two-way coupling features for the DSMC solver for the simulation of the nozzle and plume flows of a heated nitrogen thruster [12]. Also, Grabe et al. [13] focused on the means to compare the computed flow field data to experimental results using nitrogen flow emanating from a conical nozzle. While Shershnev et al. [14] conducted numerical simulations of near field of an argon plume gas exhausting from a plane wedge-like micronozzle into vacuum using the ellipsoidal statistical model and the DSMC method, Wu et al. [15] compared experimental data with a numerical solution of the DSMC method to study the plume flow interaction of a hydrogen/oxygen thruster.

However, to the best of the author’s knowledge, up to now, based on a literature review, simultaneous comparison studies of the exhaust plume flow behavior between small monopropellant and bipropellant thrusters have not been investigated yet; however, these two thrusters are mainly used as representative propulsion systems for several spacecraft. Because these thrusters possess different characteristics of chemical species and flow properties depending on the propellants used, accurate prediction and assessment on these plume gas flow influences shall be evaluated inevitably during a spacecraft development process. Thus, the purpose of the present study intends to investigate and compare the major differences of the plume gas flow behaviors simultaneously between small monopropellant and bipropellant thrusters for the first time. To ensure a numerical efficiency, the calculation domain was composed of three different subdomains depending on the physical conditions of the plume flow. By applying the appropriate numerical methods to each subdomain sequentially, individual calculated results were used as initial boundary conditions for other methods. Consequently, the present results are expected to provide useful information on selecting the appropriate propulsion system by investigating the characteristics of highly rarefied plume flows exhausted from small monopropellant and bipropellant thrusters, which can be very helpful for actual engineers practically during the design process.

## Numerical methodology

### Compressible Navier-Stokes equations

To consider the continuum flow inside a thruster nozzle, the 2-D axisymmetric compressible N-S equation in Eq 1 is adopted in the present study. The governing equations include the continuity, momentum, energy, and turbulence equations for the gas phase in vector form as follows

$$\frac{\partial Q}{\partial t}+\frac{\partial \left(E-E_{V}\right)}{\partial z}+\frac{\partial \left(F-F_{V}\right)}{\partial r}=H$$ (1)

where *z* and *r* are the axial and radial coordinates, respectively [11]. *Q* is the conservation variables of the flowfield, *E* and *F* are the inviscid flux vectors while *E*_*v*_ and *F*_*v*_ are the viscous flux vectors in the *z* and the *r* direction as presented in Eq 2.

$$Q=\;\left(\begin{array}{c} \rho \\ \rho u\\ \rho v\\ \rho e\\ \rho k\\ \rho \omega \end{array}\right)\;E=\left(\begin{array}{c} \rho u\\ \rho u^{2}+p\\ \rho uv\\ \rho uh\\ \rho uk\\ \rho u\omega \end{array}\right)\;F=\left(\begin{array}{c} \rho v\\ \rho uv\\ \rho v^{2}+p\\ \rho vh\\ \rho vk\\ \rho v\omega \end{array}\right)$$

$$E_{V}=\left(\begin{array}{c} 0\\ \tau_{zz}\\ \tau_{zr}\\ u\tau_{zz}+v\tau_{zr}-q_{z}\\ \tau_{kz}\\ \tau_{wz} \end{array}\right)\;F_{V}=\left(\begin{array}{c} 0\\ \tau_{zr}\\ \tau_{rr}\\ u\tau_{zr}+v\tau_{rr}-q_{r}\\ \tau_{kr}\\ \tau_{wr} \end{array}\right)$$ (2)

Here, *ρ*, *u*, *v*, *e*, *h* and *p* are the density, axial and radial velocity components, total energy, enthalpy and pressure, respectively. *k* and *ω* are turbulent kinetic energy and specific dissipation rate. Also, *τ* and *q* denote shear stresses and heat fluxes [12]. And *H* is the axisymmetric source term defined as below [11].

$$H=\frac{1}{r}\left(\begin{array}{c} \rho v\\ \rho uv\\ \rho v^{2}\\ \rho vh\\ \rho vk\\ \rho v\omega \end{array}\right)+\frac{1}{r}\left(\begin{array}{c} 0\\ \tau_{zr}\\ \tau_{rr}-\tau_{\theta \theta }\\ u\tau_{zr}+v\tau_{rr}-q_{r}\\ \tau_{kr}\\ \tau_{wr} \end{array}\right)$$ (3)

Total energy, enthalpy and the pressure of flowfield are given by the following equations

$$e=\sum_{i=1}^{N_{i}}Y_{i}h_{i}-\frac{p}{\rho }+\frac{1}{2}\left(u^{2}+v^{2}\right)=\sum_{i=1}^{N_{i}}Y_{i}\int C_{p,i}\left(T_{i}\right)dT-\frac{p}{\rho }+\frac{1}{2}\left(u^{2}+v^{2}\right)$$ (4)

$$p=\rho RT\overset{N}{\underset{i=1}{\sum }}\frac{Y_{i}}{W_{i}}$$ (5)

where *Y*_*i*_ and *M*_*i*_ are the mass fraction and the molecular mass of the product gases, and *R* is the universal gas constant (8314.41 J/kmol K), respectively [11]. As the physical dimension of the low thrust rocket engine is small, the thickness of the wall boundary layer cannot be disregarded. To account for the turbulence behavior of the low Reynolds number flow in the viscous sub-layer and the high velocity flow region away from the wall together, the shear-stress transport (SST) *k*−*ω* turbulence model is used in the present study.

For an efficient calculation, the governing equation of Eq 1 is discretized and integrated over each grid cell according to the finite-volume methodology as Eq 6, and then solved using the implicit method [11].

$$\frac{1}{dt}\int_{\Omega }Qd\Omega +\int_{\Gamma }\left(Edr-Fdz\right)=\int_{\Gamma }\left(E_{V}dr-F_{V}dz\right)-\;\int_{\Omega }Hd\Omega$$ (6)

### Direct simulation Monte Carlo method

**Fig 3:**
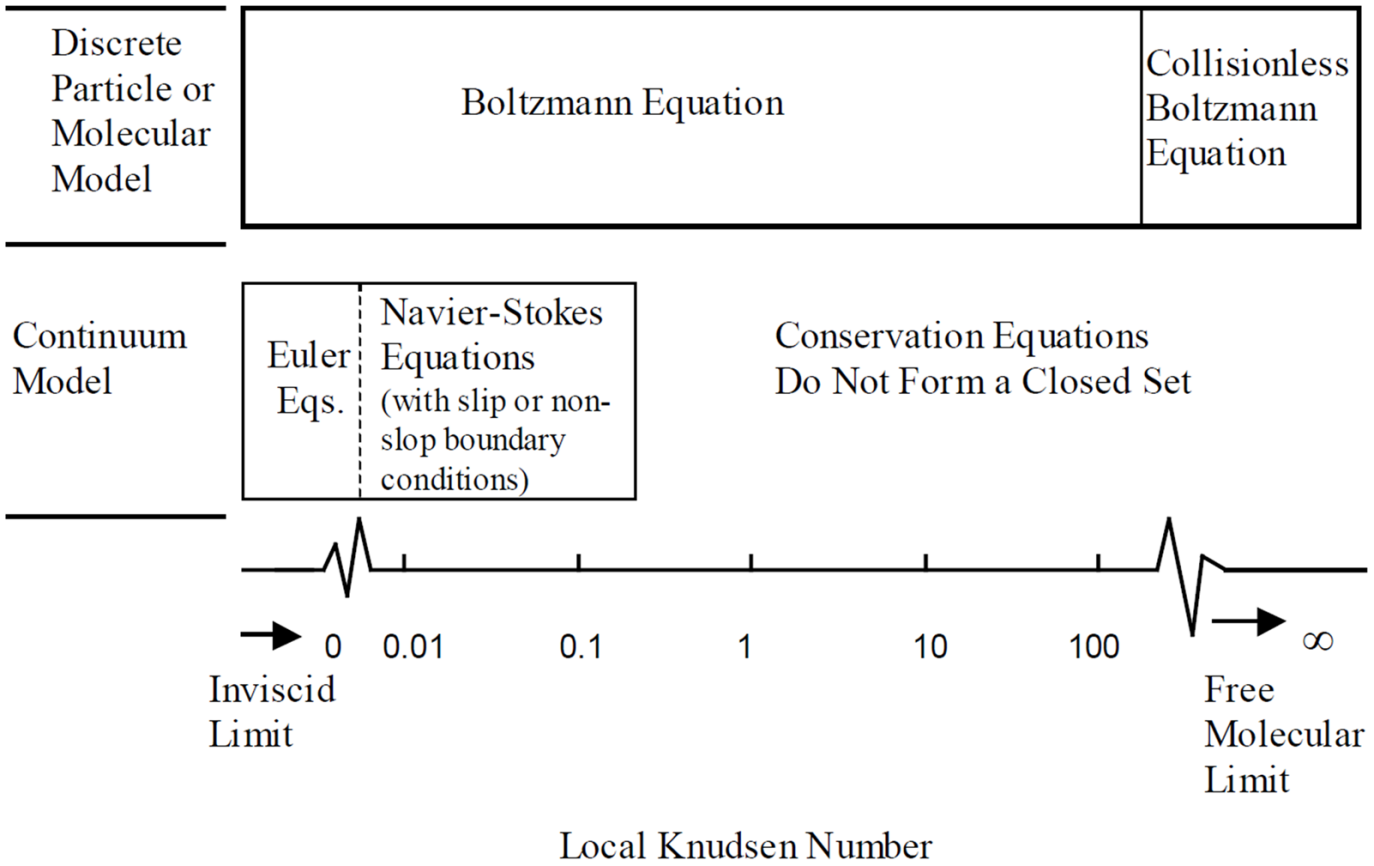
Flow regimes and valid models at different Knudsen number [5,6].

Generally, the characteristics of the flow regimes can be classified by the Knudsen number (*Kn*) which is defined as the ratio of a mean free path and a characteristic length. The application of proper fluid models and equations is divided depending on the Knudsen number range in Fig 3 [5,6]. If the Knudsen number is near or greater than one, the mean free path of a molecule is comparable to a length scale of the physical system. Thus, the continuum assumption such as the Navier-Stokes equation does not guarantee a good approximation for the high Knudsen number flow any more. In such case, the Boltzmann equation in a nonlinear form (Eq 7) should be dealt with statistical methods for the rarefied flow regime [5,6].

$$\frac{\partial }{\partial t}\left(nf\right)+\vec{v}\frac{\partial }{\partial \vec{r}}\left(nf\right)+\vec{F}\;\frac{\partial }{\partial \vec{v}}\left(nf\right)=\int_{-\infty }^{\infty }\int_{0}^{4\pi }n^{2}\left(f^{*}f_{1}^{*}-ff_{1}\right)v_{r}\sigma d\Omega \;d\vec{v_{1}}$$ (7)

Here, *n*, $\vec{\text{v}}$, $\vec{r}$, *f* and *v*_*r*_ are number density, velocity vector, position vector, probability density function and relative velocity of molecules, respectively. Also, $\vec{F}$, $d\vec{v_{1}}$, *dΩ* and *σ* are external force vector, molecules of class with velocity *v*_*1*_, elementary solid angle and collision cross section [5,6]. Among various methods, the Direct Simulation Monte Carlo (DSMC) method originally developed by Bird in 1960s [5,6] can be considered as the most effective technique for solving the nonlinear Boltzmann equation for the rarefied flow modeling. The DSMC is a direct particle simulation method based on kinetic theory and it uses a statistical method in which the number of representative simulated molecules is traced in space and time which models the physics of the real gas [5,6]. Thus, although the DSMC method generally requires a longer computational time than conventional continuum flow models, the present study uses the two dimensional axisymmetric DSMC code to describe the exhaust plume gas behaviors in the vacuum condition because conventional computational fluid dynamic schemes such as FDM (Finite Difference Method) and FVM (Finite Volume Method) do not predict accurate flow behaviors reasonably for the high vacuum regime. The present study uses the two dimensional axisymmetric DSMC code to describe the expanding low thrust plume gas flow into the vacuum condition. The variable hard sphere (VHS) model [5] is used as the intermolecular-collision model and the no-time counter (NTC) method is for the collision sampling technique [5]. The Larsen-Borgnakke model [16] is employed to redistribute the translational and the internal energy exchange between the colliding molecules. Because the overall plume temperature far from the nozzle exit is not so high, only the rotational mode is considered while the chemical reaction and vibrational mode excitation are neglected.

## Results and discussion

To ensure efficient numerical calculations such as the computational time and accuracy, the whole physical domain was divided into three different subdomains depending on the flow conditions as follows: a stagnation flow in a combustion chamber, a continuum flow regime inside a nozzle, and a rarefied plume gas flow in a vacuum space environment, respectively. And then appropriate numerical methods were combined and applied to each subdomain sequentially so that individual calculated results could be used as boundary conditions for other methods.

### Thermodynamic properties in thruster chamber

At first, the chemical equilibrium reactions of the monopropellant and the bipropellant in the combustion chamber are calculated individually to estimate the composition of the chemical species of the plume gas flow and its highest combustion temperature at the stagnation condition. Here, hydrazine (*N*_*2*_*H*_*4*_) and a combination of monomethylhydrazine (MMH, *CH*_*3*_*N*_*2*_*H*_*3*_) and nitrogen tetroxide (NTO, *N*_*2*_*O*_*4*_) are chosen for the monopropellant and the bipropellant in the present study because they are typically used for space propulsion application.

In case of hydrazine, thrust is provided by the catalytic decomposition and the final combustion product gases consist of three main species, *H*_*2*_, *N*_*2*_, and *NH*_*3*_ following overall chemical equilibrium process in Eq 8 [11,17].

$$N_{2}H_{4}\to \frac{4}{3}\left(1-f\right)NH_{3}+\frac{1}{3}\left(1+2f\right)N_{2}+2fH_{2}$$ (8)

f=[1649−T]/782 (9)

where *f* represents for the extent of ammonia dissociation at given temperature [11,17]

For the bipropellant thruster, the more complicated combustion process between the fuel and the oxidizer are involved. Overall chemical equilibrium reaction of MMH and NTO can be defined in Eq 10, where *α* is Stoichiometric coefficient and *n*_*pi*_ is the mole number of each product species [18,19].

$$4\alpha CH_{3}N_{2}H_{3}+5N_{2}O_{4}\to n_{p1}CO_{2}+n_{p2}H_{2}O+n_{p3}O_{2}+n_{p4}N_{2}+n_{p5}NO+n_{p6}CO+n_{p7}OH+n_{p8}H_{2}+n_{p9}O+n_{p10}H+n_{p11}N+n_{p12}NO_{2}+n_{p13}H_{2}O_{2}+n_{p14}HO_{2}+n_{p15}HNO$$ (10)

To solve Eq 10, the following nonlinear equations based on the mass conservation equations of the major elements (*C*, *O*, *H*, and *N*) and the equilibrium constants of eleven elementary equilibrium equations are calculated numerically at the same time [18,19].

$$n_{p1}+n_{p6}-4\alpha =0$$

$$2n_{p2}+n_{p7}+2n_{p8}+n_{p10}-24\alpha =0$$

$$2n_{p1}+n_{p2}+2n_{p3}+n_{p5}+n_{p6}+n_{p7}+n_{p9}+2n_{p12}+2n_{p13}+2n_{p14}+n_{p15}-20=0$$

$$2n_{p4}+n_{p5}+n_{p11}+n_{p12}+n_{p15}-8\alpha -10=0$$

$$n_{p5}^{2}-K_{p7}n_{p4}n_{p3}=0$$

$$n_{p6}^{2}n_{p3}P_{r}-K_{p1}n_{p1}^{2}n_{t}=0$$

$$n_{p7}^{2}n_{p8}P_{r}-K_{p2}n_{p2}^{2}n_{t}=0$$

$$n_{p8}^{2}n_{p3}P_{r}-K_{p3}n_{p2}^{2}n_{t}=0$$

$$n_{p9}^{2}P_{r}-K_{p5}n_{p3}n_{t}=0$$

$$n_{p10}^{2}P_{r}-K_{p4}n_{p8}n_{t}=0$$

$$n_{p11}^{2}P_{r}-K_{p6}n_{p4}n_{t}=0$$

$$n_{p12}^{2}n_{t}-K_{p8}n_{p4}n_{p3}^{2}P_{r}=0$$

$$n_{p13}n_{t}-K_{p9}n_{p8}n_{p3}P_{r}=0$$

$$n_{p14}^{2}n_{t}-K_{p10}n_{p8}n_{p3}^{2}P_{r}=0$$

$$n_{p15}^{2}n_{t}-K_{p11}n_{p4}n_{p8}n_{p3}P_{r}=0$$

$$n_{p1}+n_{p2}+n_{p3}+n_{p4}+n_{p5}+n_{p6}+n_{p7}+n_{p8}+n_{p9}+n_{p10}+n_{p11}+n_{p12}+n_{p13}\;+n_{p14}+n_{p15}-n_{t}=0$$ (11)

Here, *n*_*t*_ is total number of product gas moles and *K*_*pi*_ is the equilibrium constant defined by *G*, *h*, *S* which are Gibbs free energy, enthalpy, and entropy under standard condition in Eqs 12 and 13, respectively.

$$n_{t}=\sum_{i}n_{pi}$$ (12)

$$K_{pi}=exp^{-\left(\frac{\Delta G}{RT}\right)}$$ (13)

ΔG=Δh−TΔS (14)

The thermodynamic parameters of specific heat capacities of gases (*C*_*p*_), enthalpies (*h*), and entropies (*S*) are determined based on polynomials of temperature [20].

$$\frac{C_{p}}{R}=a_{1}+a_{2}T+a_{3}T^{2}+a_{4}T^{3}+a_{5}T^{4}$$ (15)

$$\frac{H}{RT}=a_{1}+\frac{a_{2}}{2}T+\frac{a_{3}}{3}T^{2}+\frac{a_{4}}{4}T^{3}+\frac{a^{5}}{5}T^{4}+\frac{a_{6}}{T}$$ (16)

$$\frac{S}{R}=a_{1}lnT+a_{2}T+\frac{a_{3}}{2}T^{2}+\frac{a_{4}}{3}T^{3}+\frac{a_{5}}{4}T^{4}+a_{7}$$ (17)

When the entire mole numbers of each product species are determined, adiabatic flame temperature can be predicted by the general energy equation of the chemical equilibrium reaction in Eq 18.

$$\Delta H_{r}^{{}^{\circ}}\;=\sum_{i}n_{pi,products}\int_{298}^{T_{ad}}C_{pi}dT$$ (18)

where *ΔH*^*o*^_*r*_ is the heat of reaction under a standard condition and *T*_*ad*_ is an adiabatic flame temperature [18,19]. To calculate mole numbers of each product and the adiabatic flame temperature, Eqs 11–18 were iterated numerically by the Newton method with an initially assumed adiabatic temperature and a chamber pressure. Then, the molecular mass (*M*) and specific heat at constant pressure and specific heat ratio of the mixture of the combustion product gases can be calculated by the following equations [18,19].

$$M=\frac{\sum n_{i}M_{i}}{\sum n_{i}}$$ (19)

$$C_{p}=\frac{\sum n_{i}C_{pi}}{\sum n_{i}}$$ (20)

$$k=\frac{C_{p}}{C_{p}-R}$$ (21)

The mixture ratio by mass is calculated from the reactants of the chemical reactions as follows

$$\frac{m_{oxdizer}}{m_{fuel}}=\frac{M_{oxdizer}\times n_{oxdizer}}{M_{fuel}\times n_{fuel}}$$ (22)

where *m* is a mass and *n* is a mole number of the fuel and the oxidizer, respectively [18,19].

The final stagnation flow conditions of each propellant with the composition of the major chemical species of the plume gas flow and its highest combustion temperature are summarized in Table 1.

**Table 1:** Chemical equilibrium reaction result of hydrazine and MMH-NTO propellants.

Results	Hydrazine	MMH-NTO
**Mole fractions of combustion gas species**		
*H*_*2*_	0.35761	0.15657
*N*_*2*_	0.27152	0.30513
*NH*_*3*_	0.37087	-
*H*_*2*_*O*	-	0.32741
*CO*	-	0.13145
*CO*_*2*_	-	0.03628
Other species	-	< 0.01
**Molecular mass of gas mixture [g/mol]**	14.62	20.46
**Adiabatic flame temperature [K]**	1342.8	3087.4

### Continuum gas flowfields inside thruster nozzle

**Fig 4:**
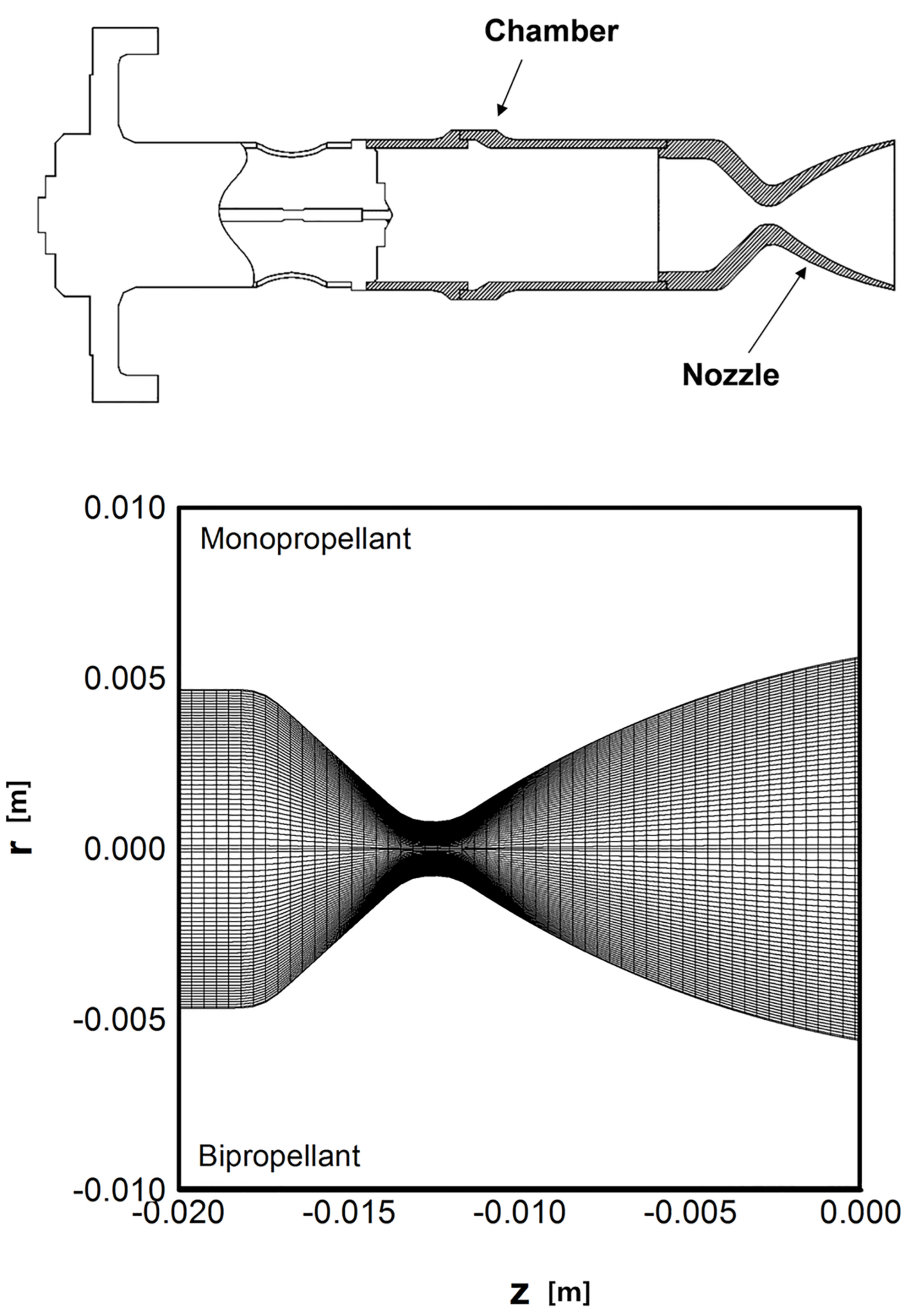
Configuration and calculation domain of thruster. (A) Configuration of small thruster [11]. (B) Calculation domain of thrusters for N-S equations.

Because plume gas flowfields in the vacuum space are influenced dominantly by the continuum nozzle flow inside the thruster, accurate prediction of the plume flow properties at the nozzle exit plane is important to specify the inflow boundary condition of the plume analysis. Thus, the computational fluid dynamics (CFD) method based on the Navier–Stokes (N–S) equations was used to simulate continuum gas flowfields inside the thruster nozzle. Fig 4A shows a brief configuration of the small thruster considered in this study with hydrazine as the monopropellant and the MMH-NTO combination as the bipropellant together. It has a conical shape nozzle with an expansion ratio of 50:1 to produce roughly a five newton thrust when the stagnation chamber pressure, *p*_*c*_, is 1.45 MPa. The computations were performed for a calculation gird with about 2,500 nodes along the nozzle axis and radius inside the thruster show in Fig 4B. For the boundary conditions, the stagnation flow data inside the chamber were used for the nozzle inlet condition, which are specified from previous chemical equilibrium reactions in Table 1. The product gas species are assumed to be a mixture of perfect gases with chemically frozen compositions during the expansion process through the nozzle. At the center line of the nozzle, the velocity component and the derivatives of all other properties in the radial direction are zero by the axisymmetric condition. The nozzle wall is assumed to have an adiabatic and no-slip condition. Additionally, an extrapolation condition is imposed at the outflow boundary.

**Fig 5:**
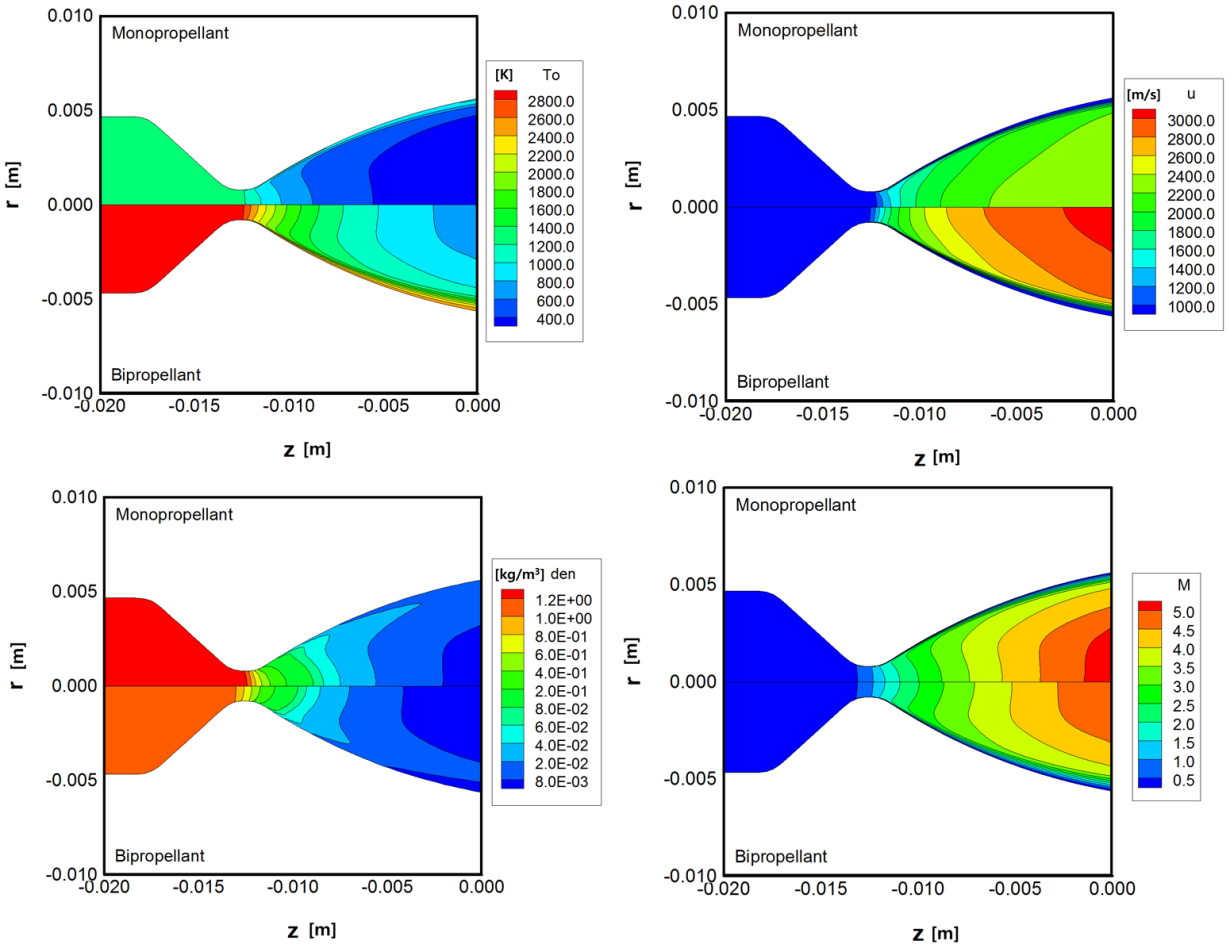
Continuum flow results inside the thruster using N-S equations. (A) Temperature [K]. (B) Axial velocity [m/s^2^]. (C) Density [kg/m^3^]. (D) Mach number.

Fig 5 presents the major analysis outcomes of the continuum nozzle flow inside the monopropellant and bipropellant thrusters. The monopropellant results occupy the upper portion and the bipropellant results are shown in the lower portion of the given figures. Because the adiabatic stagnation temperature of the bipropellant MMH-NTO is above 3,000 K initially in the chamber, Fig 5A shows that the whole gas flow under the expansion process still maintains much higher temperature levels over the entire nozzle region than that of the monopropellant for which the decomposition temperature is about 1,300 K. For example, the analysis predicts temperatures at the center of the nozzle exit plane to be about 690 K for the bipropellant and 260 K for the monopropellant, respectively. Additionally, it can be deduced that an even higher velocity profile will be estimated in the axial direction for the bipropellant thruster shown in Fig 5B because the exhaust velocity of the nozzle increases proportional to the chamber temperature following rocket performance theory. On the other hand, a denser gas flow is produced from the monopropellant decomposition process and spreads all over the nozzle domain shown in Fig 5C in contrast to the temperature distribution because the internal nozzle flow is assumed to obey the perfect gas law in Eq 5, which describes an inverse relation between the density and the temperature at a given pressure. Moreover, from the Mach number result in Fig 5D, it was found that a supersonic gas flow starts to develop through the nozzle throat and is accelerated above Mach number 5 as it approaches toward the nozzle exit. Although the axial velocity component of the bipropellant combustion gas is much faster, the Mach number of the monopropellant gas is predicted to be slightly higher than that of the bipropellant result at the core of the nozzle exit because the Mach number is defined as a function of the reciprocal of a square root of the gas temperature. The temperature of the monopropellant gas is estimated to be about 400 K lower than that of the bipropellant gas.

**Fig 6:**
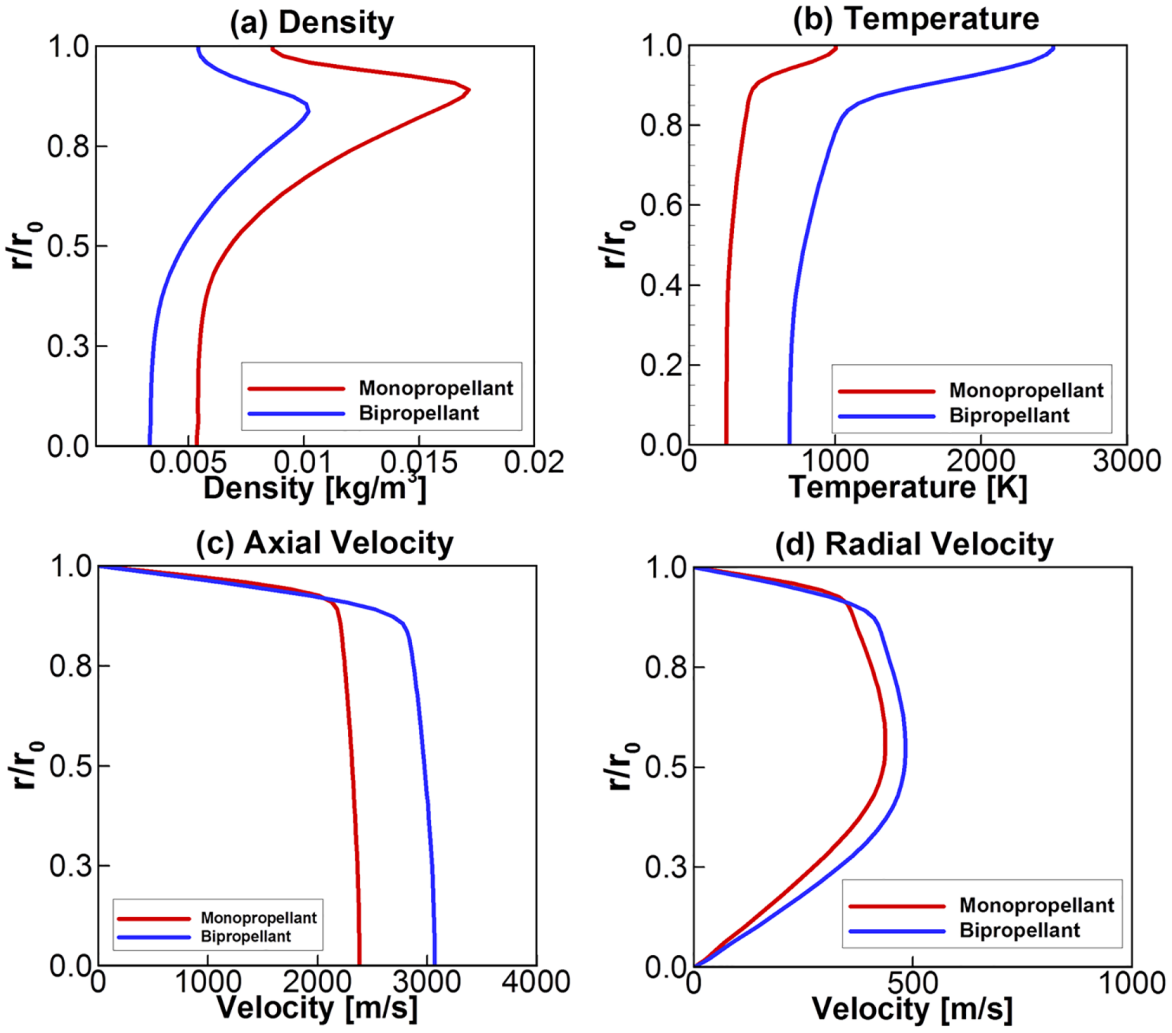
Exhaust plume flow properties at the thruster nozzle exit plane.

The final combustion gas properties at the nozzle exit plane are summarized in Fig 6 including the density, temperature, and two velocity components. Closer to the nozzle wall, large variations in the flow properties are observed in the given profiles across the compressible boundary layer region. For the given chamber stagnation conditions, the numerical solutions predict a thrust of 4.998 and 4.993 N for the monopropellant and the bipropellant cases, respectively.

### Rarefied plume gas flow in vacuum space environment

**Fig 7:**
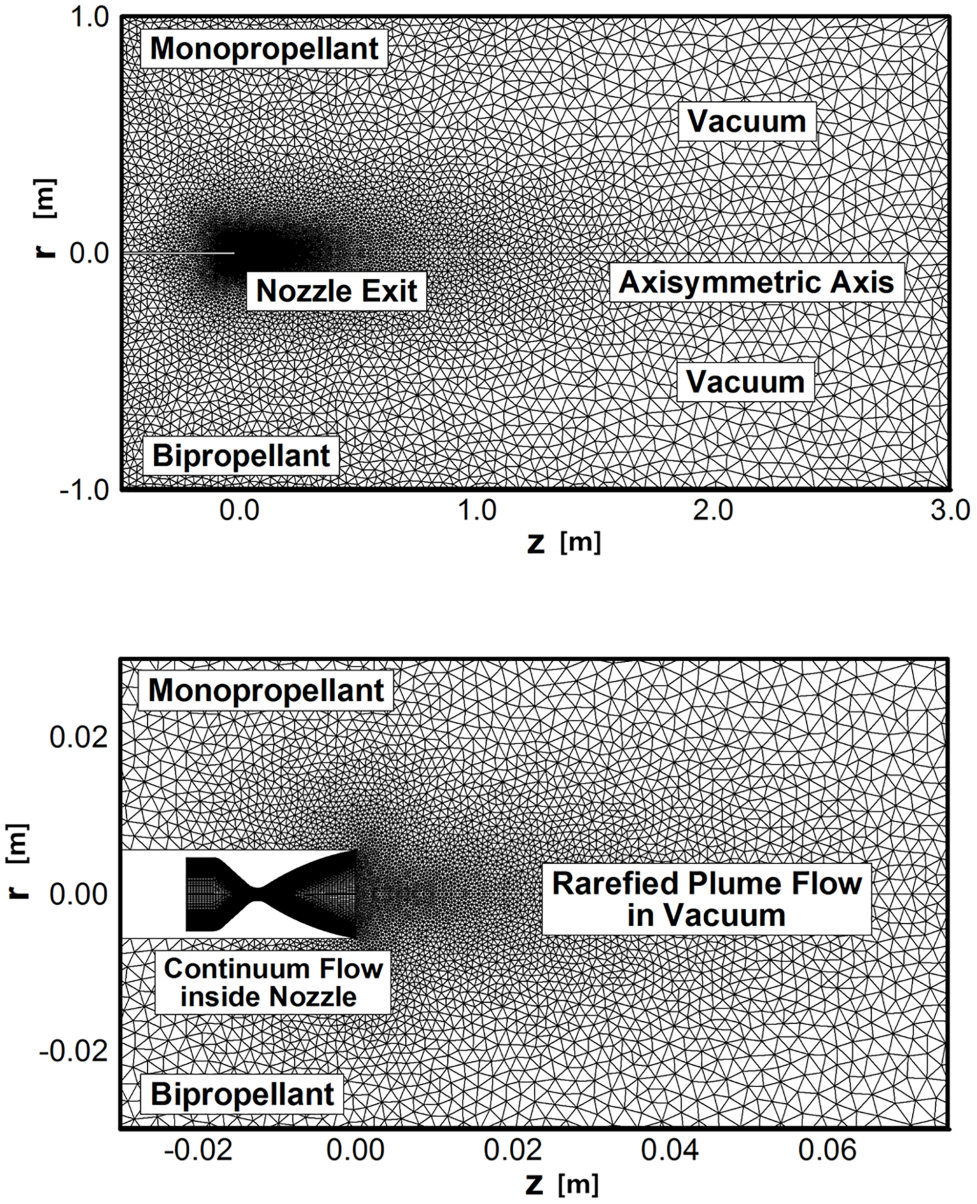
Calculation domain of plume gas flow for DSMC method. (A) Entire calculation domain. (B) Detailed domain adjacent to nozzle exit.

Together with the ten gas mixture composition including Table 1, the continuum flowfield results obtained from the N-S equations were applied as inlet conditions at the nozzle exit for the DSMC simulation of the plume flow in the vacuum. Fig 7 shows the calculation domain and boundary conditions for the DSMC method used in this study. The center of the nozzle exit plane is located at the point (0,0), and the size of the calculation domain is 3 m in the forward axial direction and 1 m in the radial direction to compare the plume expansion phenomena widely in the vacuum region. Additionally, -0.5 m backward axial region was considered to investigate the plume backflow generated from the boundary layer effect around the nozzle lip. The entire calculation domain is assumed to be a vacuum condition, while the axisymmetry condition is applied to the bottom *r* = 0 axis. The domain consisted of about 7,100 nodes and 13,800 cells with triangular grids. Almost 250,000 particles were generated when a steady state converged solution was achieved. During the DSMC calculation, the plume flowfield was sampled every 30 time steps for 10,000 iterations.

**Fig 8:**
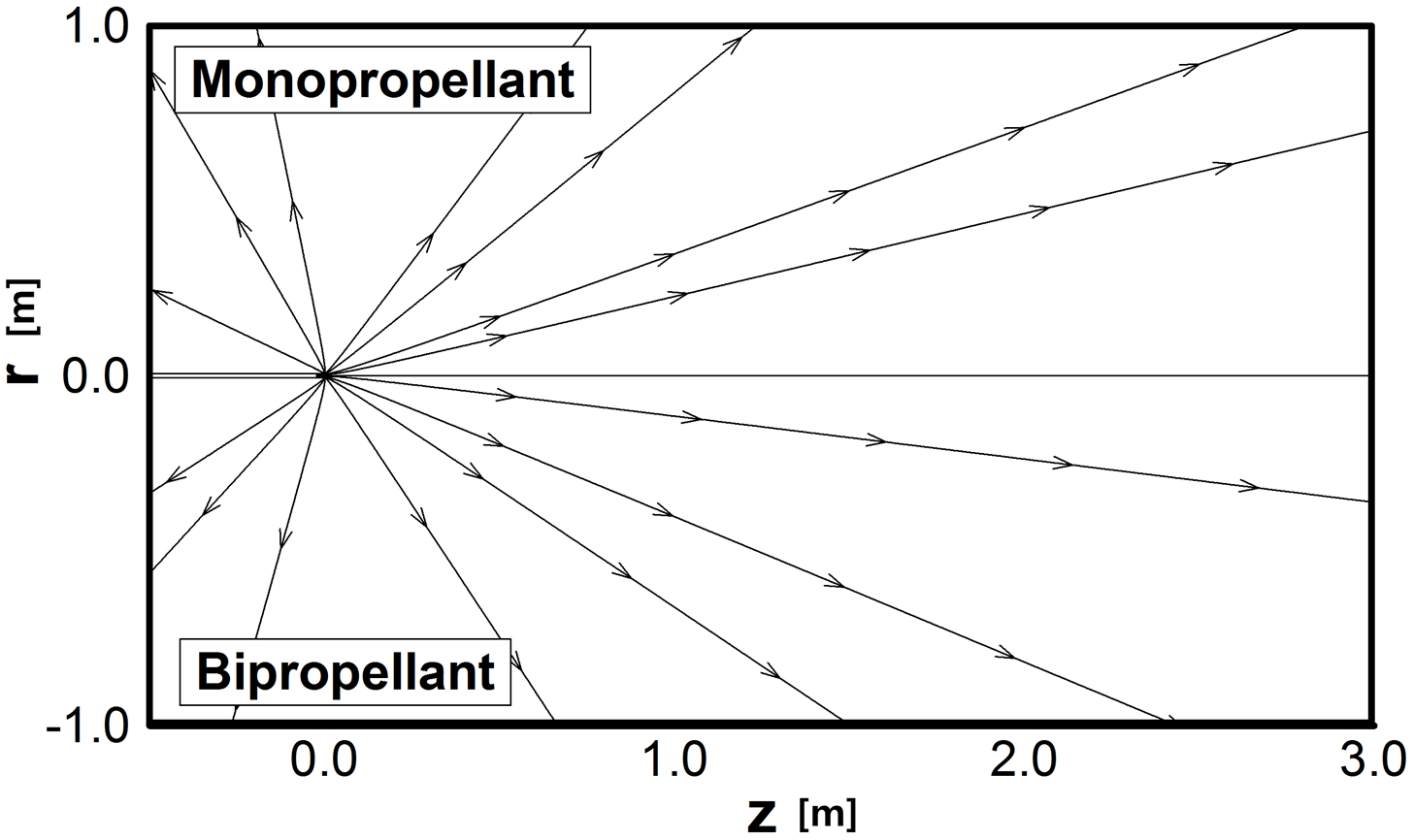
Comparison of velocity streamlines of plume gas flows.

**Fig 9:**
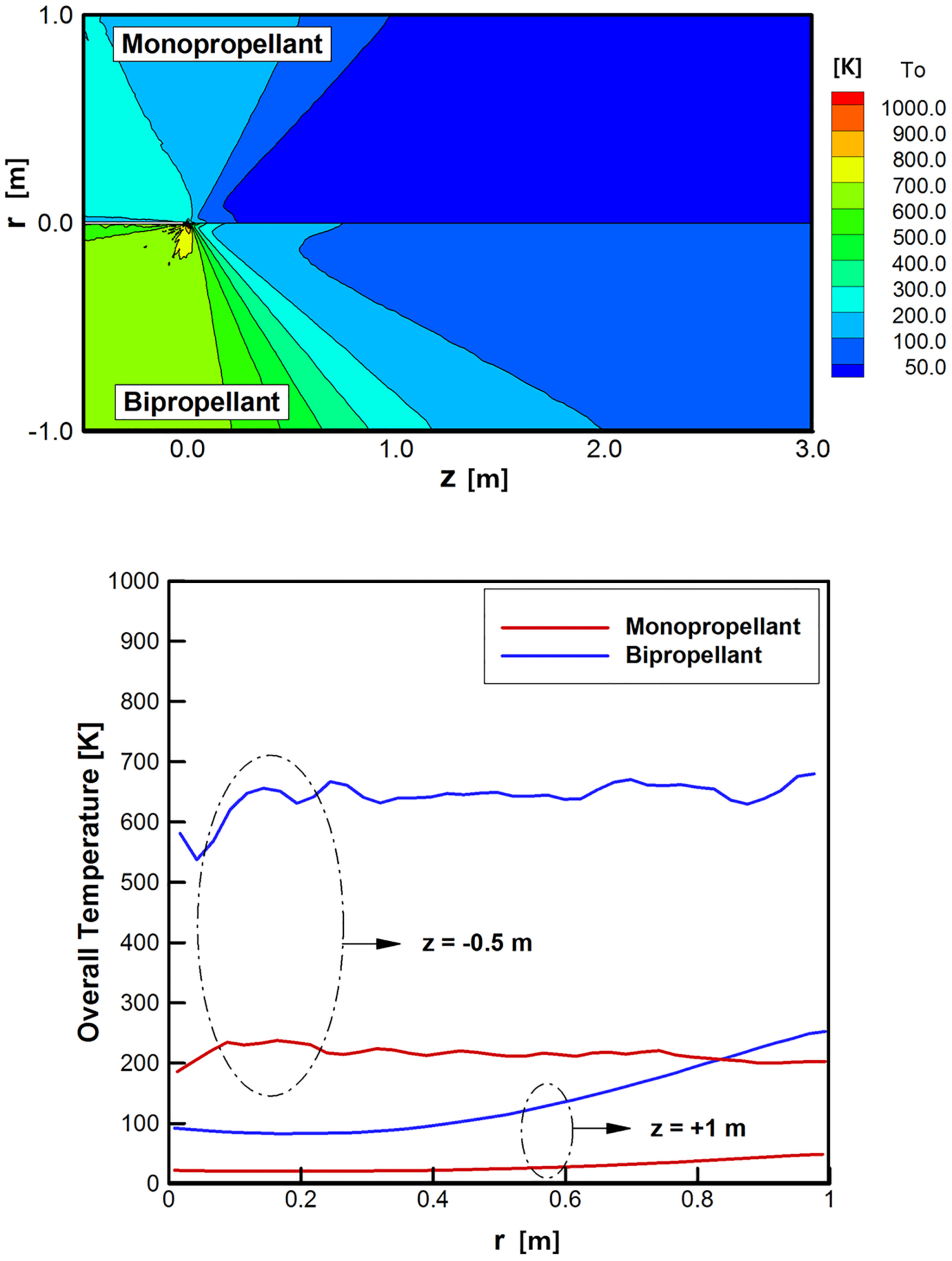
Comparison of overall temperature distributions of plume gas flows [K]. (A) Overall temperature distributions over entire calculation domain. (B) Overall temperature distributions at *z* = +1 m and *z* = -0.5 m.

**Fig 10:**
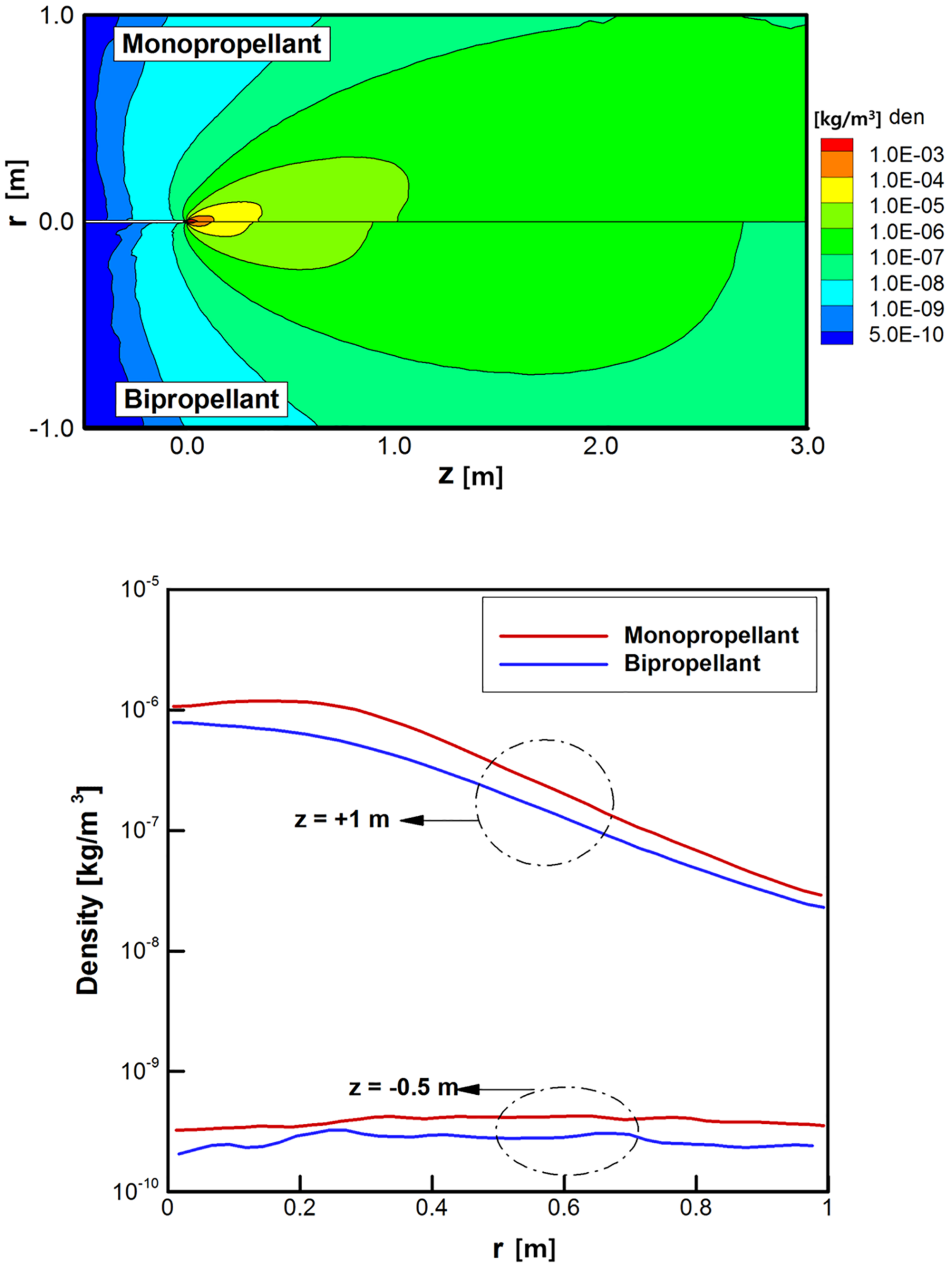
Comparison of density distributions of plume gas flows [kg/m^3^]. (A) Density distributions over entire calculation domain. (B) Density distributions at *z* = +1 m and *z* = -0.5 m.

Major differences were examined in the plume gas flowfield between the monopropellant hydrazine and bipropellant MMH-NTO combination under the vacuum condition, and Figs 8–10 show the major analysis results with the upper-half region for the monopropellant and the lower-half region for the bipropellant, respectively. Total three and fifteen gas species are considered for the monopropellant and the bipropellant plume flow, respectively. First, it is clearly observed from Fig 8 that some amount of ejected plume flow turns suddenly at an angle larger than 90° around the nozzle lip due to viscous boundary layer effects when it comes out into the vacuum condition. Both hydrazine and MMH-NTO plume gases expand in such a similar form into the thruster backflow region, which may cause various plume impingement effects directly on the spacecraft surfaces. In addition, the difference of the overall temperatures between the hydrazine and MMH-NTO plume flow gases are compared in Fig 9A. The present DSMC method predicts that the higher temperature plume gas of MMH-NTO spreads over the whole calculation domain based on the temperature profile at the nozzle exit plane in Fig 6. This indicates that the amount of thermal energy released from the chemical reactions of the propellant is dominantly dependent on the adiabatic temperature inside the thruster chamber and also it can affect the exhaust plume gas flow expanding through the nozzle. For further comparison, the overall temperature variations are plotted in Fig 9B following a radial axis direction at *z* = +1 m and -0.5 m, respectively. In the case of the hydrazine plume, its temperature decreases gradually about from 260 K at the center of the nozzle exit to 20 K at *z* = +1 m following the forward axial direction, while the temperature of the MMH-NTO plume gas varies between 690 K at the center of the nozzle exit and 83 K at *z* = +1 m. Especially, more severe temperature deviations are observed in the backflow region in which the MMH-NTO plume gas expands from 2,054 K at the nozzle lip and 537 K at *z* = -0.5 m whereas the hydrazine plume gas falls from 790 K at the nozzle lip to 185 K at *z* = -0.5 m. Therefore, a possibility is predicted that a more excessive thermal load may be transferred to the spacecraft surfaces when the MMH-NTO bipropellant thruster is used because it ejects hotter plume gas particles than that of the monopropellant thruster. In contrast to the overall temperature distributions, Fig 10A illustrates that the hydrazine plume gas flow is predicted to spread more densely all over the calculation domain including the backflow region than that of the MMH-NTO gas because a higher density profile is initially applied at the inflow condition of the DSMC method based on the continuum flow results inside the thruster nozzle. For further comparison, the density variations are shown in Fig 10B in the radial axis direction at *z* = +1 m and -0.5 m, respectively. As the plume gas flow expands gradually in the vacuum space, the density of the gas ejected from the MMH-NOT thruster falls to below 2.3E-8 kg/m^3^ (6.8E+17 No./m^3^ for the number density) at *z* = +1 m in the forward axial direction while it remains over 2.9E-8 kg/m^3^ (1.2E+18 No./m^3^) for the hydrazine plume gas. For the backflow region at *z* = -0.5 m, a higher density of the hydrazine plume gas is also estimated to be about 3.3E-10 kg/m^3^ (1.4E+16 No./m^3^) compared to the 2.0E-10 kg/m^3^ (5.9E+15 No./m^3^) of the MMH-NTO bipropellant. Consequently, the disturbance influences due to the higher collisions of the plume backflow particles on the spacecraft surfaces may be possible to be increased when the monopropellant hydrazine thruster is used instead of the MMH-NTO thruster.

**Fig 11:**
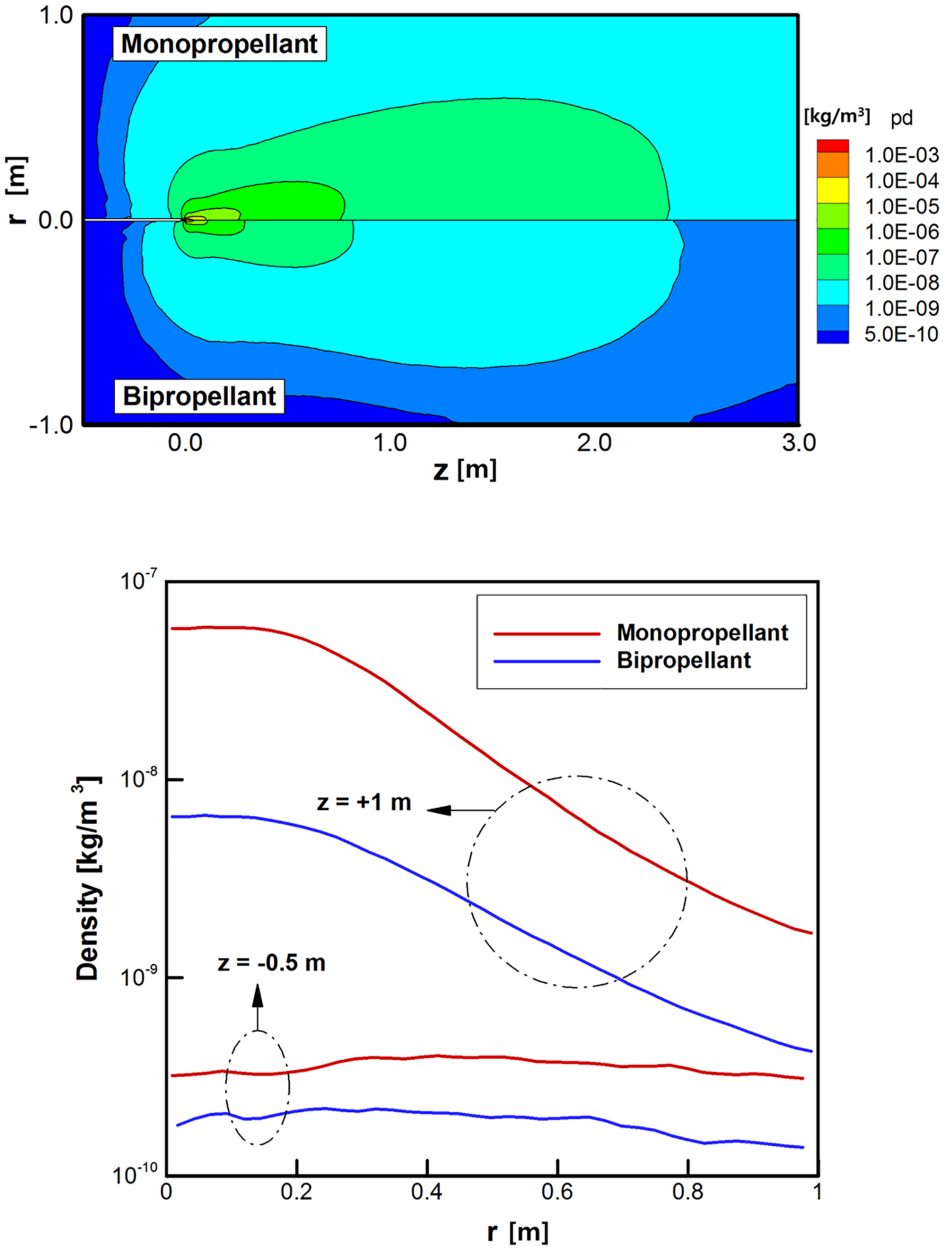
Comparison of density distributions of *H*_*2*_ species [kg/m^3^]. (A) Density distributions over entire calculation domain. (B) Density distributions at *z* = +1 m and *z* = -0.5 m.

**Fig 12:**
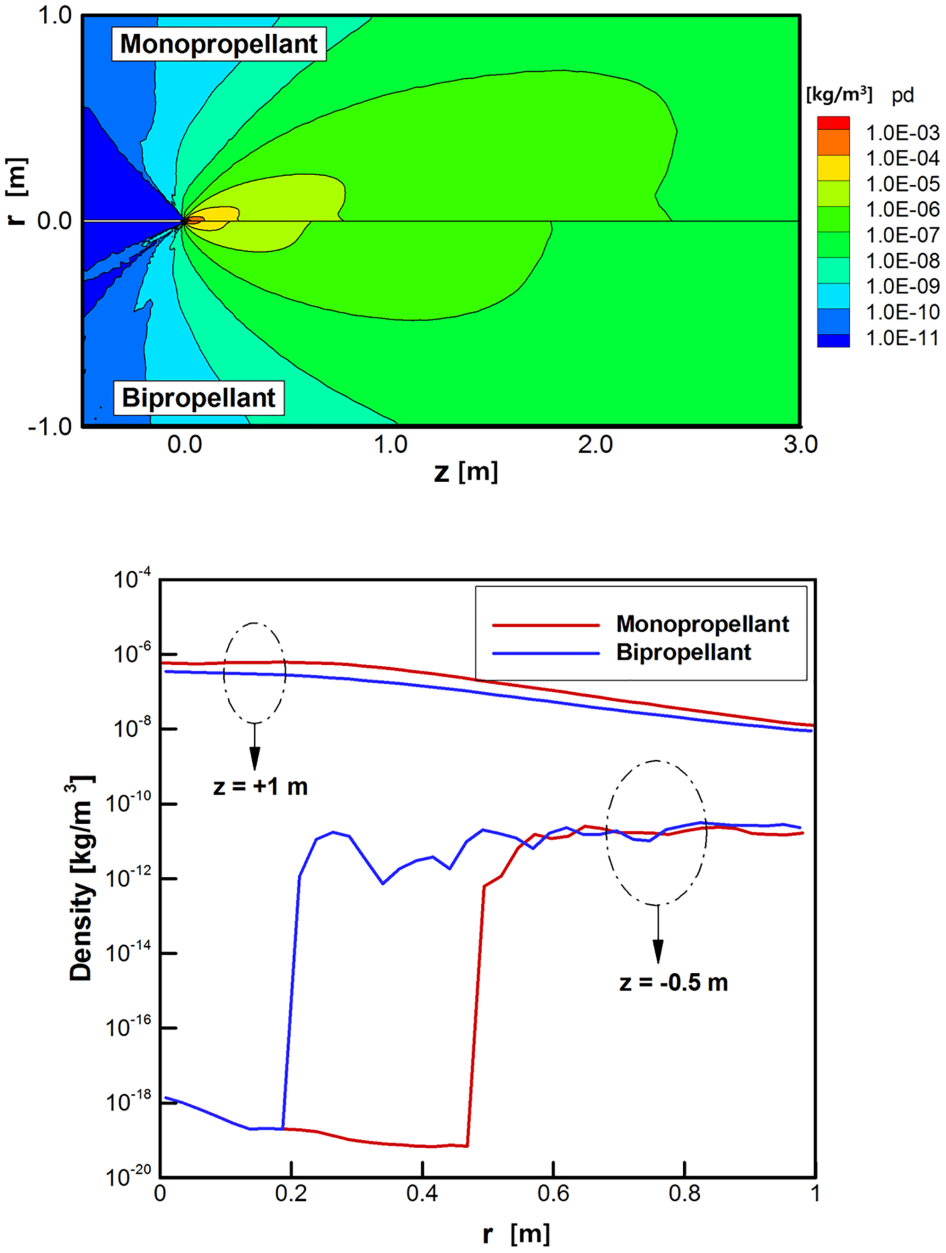
Comparison of density distributions of *N*_*2*_ species [kg/m^3^]. (A) Density distributions over entire calculation domain. (B) Density distributions at *z* = +1 m and *z* = -0.5 m.

As the final result, density distributions of the plume gas species were compared. Among the various species in Table 1, *H*_*2*_ and *N*_*2*_ were selected as the representative compositions because these are commonly included in the product gases of both propellants. Actually, the different distributions of the plume gas compositions are caused by nonequilibrium species separation due to a sudden expansion into the rarefied region. Figs 11A and 12A illustrate that the *H*_*2*_ and *N*_*2*_ species in the hydrazine plume gas are distributed more widely over the calculation domain than that of the bipropellant because of the higher density of the overall plume gas. Especially, large deviations are observed for *H*_*2*_ at the main stream (*z* = +1 m) and backflow (*z* = -0.5 m) regions in Fig 11B between the two propellants because *H*_*2*_ occupies a considerable amount of the exhaust hydrazine plume gas rather than *N*_*2*_ in Fig 12B. Thus, a possibility arose that the surface contamination by the deposition of *H*_*2*_ molecules onto the spacecraft may influence on the sensitive equipment when the monopropellant hydrazine is used as a propellant instead of the bipropellant MMH-NTO.

## Conclusions

In the present study, numerical analysis was done to investigate and compare major differences of the plume gas flow behaviors between the small monopropellant and bipropellant thrusters. To ensure an efficient numerical calculations, a combination of the chemical equilibrium reactions of the monopropellant hydrazine and bipropellant MMH-NTO, the computational fluid dynamics (CFD) method based on the Navier–Stokes (N–S) equations, and the DSMC method was used for the physical calculation domain depending on the flow conditions, which were a stagnation flow in a combustion chamber, a continuum flow regime inside a nozzle, and a rarefied plume gas flow in a vacuum space environment, respectively.

Three major differences of the plume gas flow behaviors between the two propellants were found from the present analysis results.

1. A possibility was predicted that a more excessive thermal load may be transferred to spacecraft surfaces by the plume gas impingements when the MMH-NTO bipropellant thruster is used because hotter combustion gas molecules are produced inside the thrust chamber due to a higher adiabatic temperature and also then ejected into the vacuum space region through the nozzle.
2. The monopropellant hydrazine plume gas flow was predicted to spread more densely all over the calculation domain including the backflow region than that of the MMH-NTO gas because a combustion gas with a higher density is produced inside the chamber. Consequently, the disturbance influences due to higher collisions of the plume backflow particles onto the spacecraft surfaces may be possible to be increased when the monopropellant hydrazine thruster is used instead of the MMH-NTO thruster.
3. H_2_ and N_2_ species in the hydrazine plume gas are distributed more widely over the calculation domain than that of the bipropellant because of the higher density of the overall plume gas. Especially, large deviations are observed for H_2_ at the main and the backflow regions because H_2_ occupies a considerable amount of the exhaust hydrazine plume gas. Thus, a possibility arose that the surface contamination by the deposition of H_2_ molecules onto the spacecraft may influence on the sensitive equipment when the monopropellant hydrazine thruster is used.

Consequently, the present results are expected to provide useful information on selecting the appropriate propulsion system and assessing plume flowfield behaviors in the vacuum space by investigating the characteristics of highly rarefied plume flows exhausted from small monopropellant and bipropellant thrusters, which can be very helpful for actual engineers practically during the design process. To demonstrate the present predictions of the plume behavior effects distinctly, further investigations for these two propellants are continued to compare the direct plume impingement effects on a three dimensional spacecraft configuration quantitatively using a parallel DSMC method.

## Abbreviations

*a_i_*: NASA polynomial constant
*C_p_*: specific heat at constant pressure [J/kg K]
$d\vec{v_{1}}$: molecules of class with velocity *v*_1_
*dΩ*: elementary solid angle
*e*: total energy [J/kg]
*E_v_*: viscous flux vectors in *z* direction
*F*: inviscid flux vectors in *r* direction
$\vec{F}$: external force vector
*F_v_*: viscous flux vectors in *r* direction
*f*: extent of ammonia dissociation or probability density function of molecules
*G*: Gibbs free energy
*h*: enthalpy [J/kg]
*H*: axisymmetric source term
*ΔH^o^_r_*: energy of chemical reaction under a standard condition (kJ)
*K_p_*: equilibrium constant
*M*: molecular mass [g/mol]
*m*: mass [kg]
*n*: number density [m^-3^]
*n_pi_*: number of moles of the product gas species *i* [mol]
*n_t_*: total number of moles of gas mixture [mol]
*p*: pressure [N/m^2^]
*Q_v_*: conservation variables
*q*: heat flux [W/m^2^]
*R*: gas constant [8314 J/kmol K]
*r*: radial direction distance [m]
$\vec{r}$: position vector of molecules
*S*: entropy
*T*: temperature [K]
*t*: time [sec]
*u*: axial velocity [m/s]
*v*: radial velocity [m/s]
$\vec{\text{v}}$: velocity vector of molecules
*v_r_*: relative velocity of molecules
*Y*: mass fraction
*z*: axial direction distance [m]
*α*: stoichiometric coefficient
*p*: density [kg/m^3^]
*σ*: collision cross section
*τ*: shear stress [N/m^2^]
*Ω*: control volume
*Ω_v_*: diffusion collision integral
*k*: turbulent kinetic energy [m^2^/s] or specific heat ratio
*ω*: specific dissipation rate [s^-1^]
*: post-collision value
*i*: *i*^*th*^ chemical species
*fuel*: fuel
*oxidizer*: oxidizer
*r*: radial direction
*z*: axial direction
*θ*: axisymmetric viscous source term
